# Direct Response Elements of BMP within the *PV.1A* Promoter Are Essential for Its Transcriptional Regulation during Early *Xenopus* Development

**DOI:** 10.1371/journal.pone.0022621

**Published:** 2011-08-03

**Authors:** Hyun-Shik Lee, Sung-Young Lee, Hyosang Lee, Yoo-Seok Hwang, Sang-Wook Cha, Soochul Park, Jae-Yong Lee, Jae-Bong Park, SungChan Kim, Mae Ja Park, Jaebong Kim

**Affiliations:** 1 Department of Biochemistry, College of Medicine, Hallym University, ChunCheon, Kangwon-Do, Korea; 2 Department of Anatomy, School of Medicine, Kyungpook National University, Daegu, Korea; 3 Department of Life Science, College of Natural Science, Sookmyung Women's University, Seoul, Korea; 4 School of Life Sciences, College of Natural Sciences, Kyungpook National University, Daegu, Korea; Texas A&M University, United States of America

## Abstract

*Xvent* homeobox genes encode transcription factors that repress organizer genes and are essential for dorsoventral specification during early embryogenesis in *Xenopus*. In contrast to the *Xvent-2* gene subfamily, *Xvent-1* subfamily members, including *PV.1A*, have been proposed as indirect targets of Bone Morphogenetic Protein-4 (BMP-4) signaling. Because *PV.1A* is a critical downstream mediator of, and tightly regulated by, BMP-4 signaling, we hypothesized that its promoter contains a direct BMP-4 response element to effect this transcriptional regulation. We demonstrate that direct regulation by BMP-4 is necessary for transcription of *PV.1A*: its proximal promoter contains *cis*-acting binding elements for Smads and Oaz crucial to induction in response to BMP-4 signaling. In addition to these direct *cis*-acting BMP-4 responsive elements, an indirect Xvent-2 response element and several repressive elements exist in the *PV.1A* promoter to regulate its transcription. In summary, *PV.1A* undergoes combinatorial regulation during early *Xenopus* development as both the direct target of BMP-4 signaling and as the direct and indirect target of positive and negative regulatory factors.

## Introduction

Dorsoventral patterning in developing *Xenopus* embryos is established in part by a gradient of Bone Morphogenetic Protein (BMP) signaling generated by the BMP antagonists Chordin, Noggin, and Follistatin in the extracellular space. Binding of BMPs, which effect ventralization, to antagonists prevents their interaction with their cognate receptors, leading to embryo dorsalization in overexpression studies [1], [2], [3].

In vertebrates, BMPs play critical roles in dorsoventral patterning of the early embryonic mesoderm and specification of the epidermis. In *Xenopus*, BMP-2, BMP-4, and BMP-7 ventralize the early mesoderm and negatively regulate neurogenesis [4], [5]; BMP-4 in particular is instrumental in tissue patterning and fate determination during embryonic development [6]. Intracellular BMP-4 signaling is mediated through Smad proteins, which translocate into the nucleus to activate the transcription of target genes [7], and ectopic expression of these targets, including the homeobox-containing genes *Xvent-1* [8], *PV.1* [9], *Xvent-2* [10], and *Xmsx-1* [11], recapitulates the effects of BMP-4 signals. Inhibition of BMP-4 signaling represses transcription of Xvent homeobox proteins [12], while BMP-4 overexpression induces ectopic expression of *Xvents* during early embryonic development [12], [13], [14].

*Xvent* family members can suppress dorsal fates, and promote ventral, when ectopically expressed [15]. BMP-4 signaling induces the expression of *Xvent* members that repress transcription of dorsal-specific genes [13], [14], [16] and rescue the dorsalized phenotype affected by dominant-negative Type I BMP receptor (DN-BR) [17]. The *Xvent* family comprises two subfamilies subdivided on the basis of their amino acid sequence: *Xvent-2* (*Xvent-2* [10], *Xbr-1b* [18], *Xom* [19], *Vox* [20] and *Xvent-2B* [21]) and *Xvent-1* (*Xvent-1* [8], *Xvent-1B* [21], *PV.1* [9], and *PV.1A*).

*Xvent-2* transcription is regulated by Smad1/4 and its co-activator Oaz in response to BMP-4 signaling [22]. However, the transcriptional regulation of these *Xvent* families differs. According to previous reports, the *Xvent-2*, but not the *Xvent-1* [15], family is a direct target of BMP-4 signaling [16], [22], [23]. Nevertheless, expression of *Xvent-1B*, a highly conserved *Xvent-1* paralogue, is induced indirectly by BMP-4 signaling [9].

In order to understand how BMP-4 signaling regulates the transcription of *PV.1A*, we isolated genomic DNA (gDNA) encompassing *PV.1A* and its 5′-flanking region (−2525 bp) and analyzed the regulatory elements in its promoter with regard to BMP-4 signaling during early embryonic development. The region 180-bp upstream of the major transcriptional initiation site promoted full reporter-gene activity and contained *cis*-acting elements responding directly to BMP-4 signaling. The proximal region of the promoter possessed putative binding sites for Smads and Oaz, both of which were necessary for response to BMP-4 signaling. Additionally, we determined that the promoter contains a BMP-4 response element (BRE) and an Xvent-2 response element (XRE). Furthermore, we identified negative *cis*-acting elements that responded to the dorsal-specific transcription factors AP-1 and Goosecoid in the proximal *PV.1A* promoter. These results suggest that the proximal *PV.1A* promoter contains multiple *cis*-acting elements, including direct, indirect, and negative response elements, as well as those binding transcriptional co-activators, allowing BMP-4 signals to converge and maintain tight transcriptional regulation during early embryonic development in *Xenopus*.

## Materials and Methods

### Ethics Statement

Approval from the Institutional Animal Care and Use Committee (IACUC) is not required for the experimental use of amphibians or reptiles in Korea. All members of our research group attended educational and training courses on appropriate care and usage of experimental animals. Adult *X. laevis* were entrained in 12 hr light/dark (LD 12∶12 h) cycles at 18°C in containers from the Institutes of Laboratory Animal Resources built to specifications for laboratory animal maintenance.

### DNA and RNA preparation

cDNAs encoding BMP-4, DN-BR, Smad1(3.4SA), Smad1(3SA), Oaz, dominant-negative Xvent-2 (DN-Xvent-2) [21], Goosecoid, c-Jun, and c-Fos were all subcloned into the pSP64T expression vector. *Smad1* wild-type sequence and that of its 3SA and 4SA mutants was subcloned into pSP64TEN. *Smad1*(3.4SA) was generated from *Smad1*(3SA) by replacement of the linker region of *Smad1*(4SA). *Smad2* and *Smad2*(EPSM) were subcloned into the pCS2+ vector [24]. Each vector was linearized with the appropriate restriction enzyme and used for *in vitro* transcription using a MEGAscript kit according to manufacturer's instructions (Ambion, Austin, TX). Synthetic RNAs were quantitated via ethidium bromide staining by comparison to a standard RNA control (Invitrogen, Carlsbad, CA).

### Cloning of *PV.1A* genomic DNA

*PV*.*1A* genomic DNA (gDNA) was isolated by screening a *Xenopus* muscle gDNA library (Clontech) with a PCR-amplified cDNA probe corresponding to the segment from +48 to +215 bp of *Xenopus PV.1A* cDNA. PCR primers were (upstream) 5′-CCTTCAGCATGGTTCAACAG-3′ and (downstream) 5′-CATCCTTTCTCCTTGGCATCTCCT-3′. Approximately 7.0×10^6^ plaque forming units (pfu) were screened using an ECL system in accordance with manufacturer's instructions (GE Healthcare Biosciences, Pittsburgh, PA) to identify putative positive clones, which were subsequently isolated and analyzed by restriction mapping and Southern blotting. A 3.8 kb DNA fragment in a positive clone was subcloned into the pBluescript SK(-) plasmid (Stratagene, Cedar Creek, TX). Both strands of DNA were sequenced by the Sanger method to confirm the identity of the clone.

### *PV.1A* promoter constructs

A 3.8 kb fragment from a positive clone containing 2.5 kb of 5′-flanking region was subcloned into the pGL-2 basic plasmid (Promega, Madison, WI); the clone was designated the -2525 construct. Serially deleted *PV.1A* promoter mutants were made from this -2525 construct by PCR amplification (Table 1). PCR conditions were as follows: 1 minute at 94°C, 1 minute at 54°C, and 1 minute at 72°C for 30 cycles. PCR amplification products were digested with *Xho*I/*Hind*III and inserted into similarly digested pGL-2 basic plasmid. A triple-repeat BMP-4-response element (BRE) was generated by annealing two complementary oligos:

**Table 1:** Primers used for serially deleted reporter gene constructs.

	Primer name	Sequences (5′ → 3′)
**Upstream primers**	-2525	AGTCCTCGAGTACCTGCAACTTACTCGC
	-399	AGTCCTCGAGCCAGTCTCCTGGTGTGACTT
	-374	AGTCCTCGAGCCAACATAAAAGGATAAAGG
	-351	AGTCCTCGAGAGAGGTTGTTCTTATTGGTG
	-330	AGTCCTCGAGGCTCAATAACAACATCAAGG
	-300	AGTCCTCGAGAACCTACATTATCTCTTTCC
	-262	AGTCCTCGAGTCTCTGCTGTCTGTCCATGGGA
	-240	AGTCCTCGAGTTCTGTGCCGGCCAATGCTAAT
	-204	AGTCCTCGAGCCTCCAATATCACAAGGTGAA
	-180	AGTCCTCGAGACTAACCTGACAGACTCACTGG
	-162	AGTCCTCGAGACTGGAGCCAGGACCAGG
	-136	AGTCCTCGAGCTACAAGTGAGAACATAA
	-103	AGTCCTCGAGTAGCCCATTCTGATAGCC
	-180 MT	AGTCCTCGAGACTAACCTGACCAACTCACTGG
	ORE(M) -180	AGTCCTCGAGTAACCTGACAGACTCACTAAAGCCAGGAC
**Downstream primer**		AGTCAAGCTTGATGGAGCCGCTGGAGTTGTG

5′-ACTAACCTGACAGACTCACTAACCTGACAGACTCACTAACCTGACAGACTC-3′ and 5′-GAGTCTGTCAGGTTAGTGAGTCTGTCAGGTTAGTGAGTCTGTCAGGTTAGT-3′ before subcloning into the pGL-2 basic plasmid.

### Embryo injection and explant culture

*Xenopus* embryos were obtained by *in vitro* fertilization after induction of female frogs with 500 units of human chorionic gonadotropin (Sigma, St. Louis, MO). RNAs were injected into the animal pole of 2-cell stage embryos; animal caps were dissected from injected embryos at stage 8.5 and incubated to stage 11 in 0.5X modified Barth's saline [0.5X MBS: 44 mM NaCl, 0.5 mM KCl, 0.35 mM CaCl_2_, 0.5 mM MgCl_2_, 2.5 mM HEPES (pH 7.8), 1.25 mM NaHCO_3_] for RT-PCR, measurement of luciferase activity, and electrophoretic mobility shift assays (EMSAs).

### RNA isolation and RT-PCR

*Bmp-4* mRNA (0.5 ng) was injected into the animal pole of 2-cell stage *Xenopus* embryos. At stage 7.5, embryos were treated with 25 µg/mL cycloheximide (CHX) in 0.5X MBS and maintained until control untreated embryos reached stage 8.5. Animal caps were then dissected from the injected embryos and incubated until stage 11 in 0.5X MBS containing 25 µg/mL CHX. Total RNA was isolated from whole embryos or animal caps using TRIzol reagent following the manufacturer's instructions (Invitrogen, Carlsbad, CA) and treated with DNase I to remove gDNA contamination. RT-PCR was performed with Superscript II (Invitrogen, Carlsbad, CA), as described by the manufacturer, with 2 µg total RNA per reaction. PCR was performed according to the following conditions: 1 minute at 94°C, 1 minute at each annealing temperature, 1 minute at 72°C; 20-28 cycles of amplification (Table 2).

**Table 2:** Primers used for PCR amplification.

Gene Name	Sequences (5′ → 3′)	Annealing Temp. (°C)	Cycles
**EF-1α**	U: CAGATTGGTGCTGGATATGCD: ACTGCCTTGATGACTCCTAG	56	20
**Xvent-2**	U: AACGGGAAATCCAAGATGGCD: TTTTGTTTGTCCTGCGGGAG	57	23
**Xvent-1**	U: TTCCCTTCAGCATGGTTCAACD: GCATCTCCTTGGCATATTTGG	57	25
**PV.1A**	U: CCTTCAGCATGGTTCAACAGD: CATCCTTCTTCCTTGGCATCTCCT	60	27
**Goosecoid**	U: ACAACTGGAAGCACTGGAD: TCTTATTCCAGAGGAACC	57	28

### Luciferase assays

Levels of luciferase reporter activity were measured by the luciferase assay system according to manufacturer's instructions (Promega, Madison, WI). Five different groups of animal caps (3 to 5 animal caps per group) were harvested and homogenized in 10 µL lysis buffer per animal cap. 10 µL animal cap homogenate were assayed with 50 µL luciferase substrate and activity determined by luminometer (EG & G Berthold, Bad Wildbad, Germany). All experiments were repeated at least three times using independently derived sample sets.

### *In vitro* translation and electrophoretic mobility shift assays

cDNAs encoding Smads 1, 3, and 4 were PCR-amplified and used as templates for *in vitro* translation. *In vitro* translated proteins were prepared using the T_N_T Quick Coupled Transcription/translation System according to manufacturer's instructions (Promega, Madison, WI). Promoter fragments or 3x-repeat oligonucleotides were amplified by annealing two complementary oligos, respectively, and labeled with α-^32^P-ATP using T4 polynucleotide kinase (Promega, Madison, WI). Labeled DNA probes were incubated with 10 µg animal cap protein extract or 1 µg *in vitro* translated protein at room temperature for 20 minutes in 10 µL binding buffer (2.5% glycerol, 5 mM MgCl_2_, 50 ng/µL Poly [dI·dC], and 0.05% NP-40). DNA/protein complexes were separated on 4% polyacrylamide gels. Competition experiments were performed using a 100-fold excess of unlabeled DNA probe.

### Site-directed mutagenesis

Mutagenesis was performed by an overlap extension PCR method using the several oligonucleotides in accordance with instructions (Table 3).

**Table 3:** Primers used for mutant gene constructs.

Mutated site	Name	Primer name	Sequences (5′ → 3′)
XRE	XRE(M)	M-1	ATGCTTACGTTCCTTAGCCCATTC
		M-2	GAATGGGCTAAGGAACGTAAGCAT
		-180	AGTCCTCGAGACTAACCTGACAGACTCACTGG
		Rev.	ACATCACTGTTCCAGGAAGGCAGG

### Nucleotide sequence accession number

The *PV.1A* (accession number; AF133122) cDNA sequence has been submitted to GenBank.

### Statistical analysis

The data are presented as mean and standard deviation of measurements from at least three separate and independent experiments. Differences were considered significant at *P*<0.05.

## Results

### Concomitant overexpression of Xvent-2 and GATA-2 is not sufficient for complete activation of *PV.1A* transcription in the absence of direct BMP-4 signaling

Previous work posits that, unlike Xvent-2, Xvent-1 subfamily members are not under the direct transcriptional control of BMP-4 [21]. Moreover, expression of Xvent-2 and GATA-2 is sufficient for transcription, in the presence of cycloheximide (CHX), of *Xvent-1* on the ventral side of the embryo [15]. However, in the absence of BMP-4, we observed abolition of *PV.1A* transcription despite continuing transcription of *Xvent-2* and *GATA-2*, indicating that transcription of *Xvent-1 *subfamily members relies to a greater degree on BMP-4 signaling than that of its direct targets *Xvent-2* and *GATA-2*. Furthermore, the increases in *Xvent-2* and *GATA-2* transcription were less sensitive to BMP-4 overexpression than that of *PV.1A*, in contrast to our expectation (data not shown).

**Figure 1:**
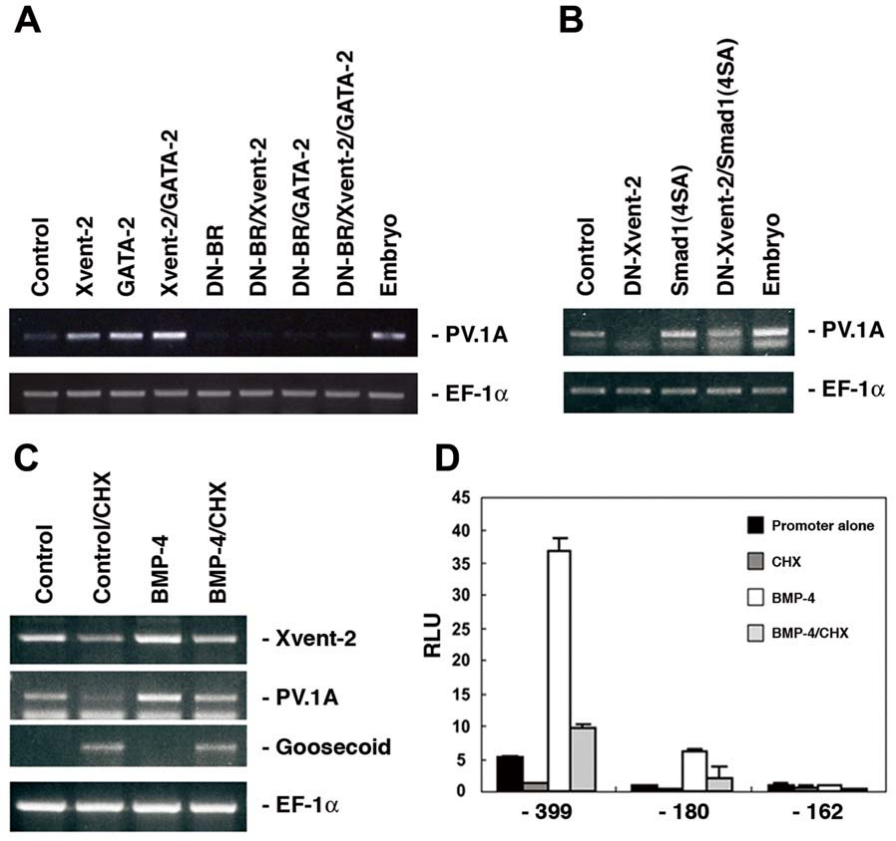
*PV.1A* is a direct target of BMP-4 signaling. (A, B) Animal caps injected with the indicated RNAs (0.5 ng/embryo) were dissected from stage 8.5 embryos and incubated until stage 11. Total RNA was isolated for RT-PCR and assayed to evaluate *PV.1A* expression. (C, D) *Xenopus* embryo animal poles were injected at the 2-cell stage with the specified promoter constructs (20 pg/embryo) in the presence or absence of BMP-4 (0.5 ng/embryo). At stage 7.5, embryos were treated with 25 µg/mL CHX in 0.5X MBS until control embryos reached stage 8.5. Animal caps were then dissected from the injected embryos and incubated to stage 11 in 0.5X MBS for RT-PCR analysis (C) or measurement of luciferase activity (D). Luciferase activity was measured as described in Materials and Methods. Gene expression was normalized to *EF-1α* transcription. Experiments were repeated three times using independent sample sets. Data are shown as mean ± SD.

We examined the necessity of BMP-4 signaling in the regulation of *PV.1A* transcription, finding that BMP-4 contributes both directly and indirectly to the process (Fig. 1A). We first assessed the capacity of Xvent-2 and GATA-2 to rescue the dramatic repression of *PV.1A* affected by dominant-negative Type I BMP receptor (DN-BR) in animal cap explants (Fig. 1A); co-injection of mRNAs encoding these factors could not reverse this repression. This suggests that although *PV.1A* transcription is augmented by Xvent-2 and GATA-2, its transcriptional activation is in fact dependent on functional BMP signaling. Conversely, the reduction in *PV.1A* transcription occurring in the presence of dominant-negative Xvent-2 (DN-Xvent-2) was mitigated by co-injection of a constitutively active *Smad1* mutant (*Smad1*(4SA), with four serine residues altered to alanine at the inhibitory phosphorylation sites in its linker region; Fig. 1B).

We next wished to examine the effects of BMP-4 signaling on *PV.1A* expression in animal cap explants under CHX treatment. As shown in Figure 1C, prior to CHX addition, the synthesis of BMP-4 led to activation of *Xvent-2* and *PV.1A* transcription (Fig. 1C). As a control, transcriptional induction of *Goosecoid* was examined to confirm that CHX could block protein synthesis. Notably, *Goosecoid* was expressed even in the presence of BMP-4 in CHX-treated animal cap explants; its expression is known to be negatively regulated by repressor protein(s) induced by BMP signaling [25]. Further, in reporter assays using various deletion constructs of the *PV.1A* promoter, 2 of 3 reporter activities were positively affected by BMP-4 in CHX-treated animal cap explants (Fig. 1D). The results indicate that *PV.1A* is in part a direct target of BMP-4 signaling.

### Isolation of the *PV.1A* 5′-flanking region

The injection of *Bmp-4* mRNAs enhances embryonic expression of *Xvent* family members [8], [9], [10]. To analyze the mechanics of *PV.1A* transcription in regard to BMP-4 regulation, we cloned and characterized its genomic locus. A 3.8-kb fragment containing the *PV.1A* 5′-flanking region and a part of its coding region was cloned from a gDNA library (Genbank accession number; AF133122). The fragment contained the promoter and sequence upstream to -2525 bp, including a TATA-like element proximal to the transcriptional start-site (TATAA). The proximal sequence of our isolated *PV.1A* promoter was nearly identical to that of *Xvent-1B*, and differed from those of *Xbr*-*1a*/*Xvent-2* and *Xvent-2B*; additionally, it contained putative BMP-4 and Oaz-response elements (BREs and OREs, respectively) at positions similar to the sites found in the *Xbr-1a*/*Xvent-2* and *Xvent-2B* promoters (Fig. S1A).

### Identification of a positive-regulatory element in the 5′-flanking region of *PV.1A*

**Figure 2:**
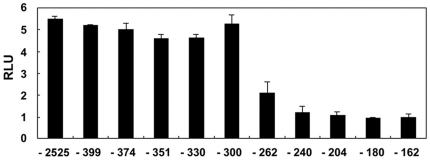
Identification of a positive-regulatory element in the 5′-flanking region of *PV.1A*. A putative element positively regulating *PV.1A* transcription was identified between −300 and −262 bp from the transcriptional start site. 2-cell stage embryos were injected at their animal poles with 20 pg of serially deleted *PV.1A* promoter constructs driving luciferase reporter transcription. Animal caps were dissected at stage 8.5 and incubated until stage 11 in 0.5X MBS for measurement of luciferase activity as described in Materials and Methods. Experiments were repeated three times using independent sample sets. Data are shown as mean ± SD.

Our detection of putative BREs and OREs, believed to be under direct regulation by the BMP-4 signaling pathway, in the *PV.1A* 5′ genomic flanking region led us to probe the nature of the *cis*-acting elements that can evoke *PV.1A* transcription. We created serial deletions of 5′-flanking sequences (Fig. S1B), and compared promoter activities among these mutants after the injection of equimolar concentrations of each reporter construct. Promoter activities were measured via luciferase assay in stage 11 animal caps. Deletion of *PV.1A* from 2525 bp to 300 bp (-300 construct) upstream of the major transcriptional initiation site did not alter promoter activity. However, the -262 construct had only half the capacity for transcriptional induction of the -300 construct (Fig. 2), indicating that the region between −300 and −262 bp contains an element that positively influences *PV.1A* promoter activity.

### Identification of a BMP-4 response element in the 5′-flanking region of *PV.1A*

**Figure 3:**
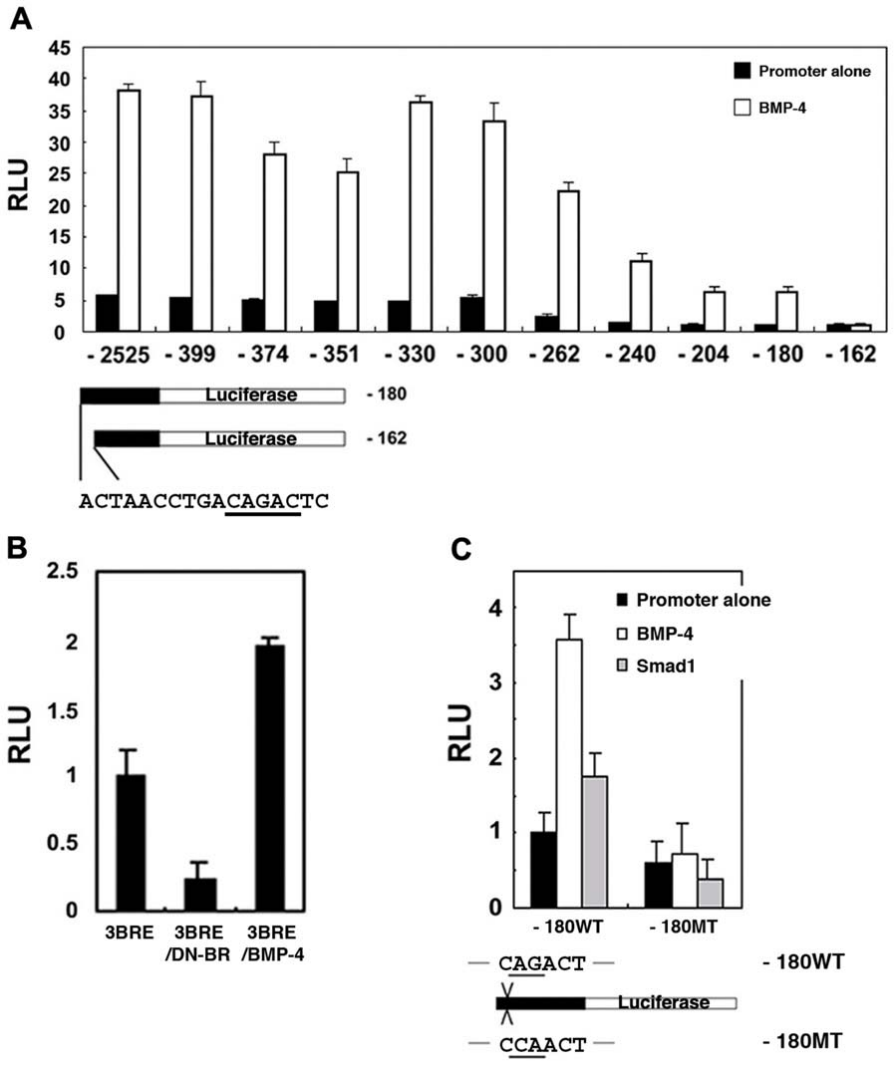
Identification of a BMP-4-response element in the 5′-flanking region of *PV.1A*. (A) Serially deleted promoter constructs were injected (20 pg/embryo) with or without *BMP-4* (0.5 ng/embryo) into 2-cell stage embryos. Animal caps were dissected from injected embryos at stage 8.5 and incubated until stage 11 in 0.5X MBS for measurement of luciferase activity. A putative BRE was detected between −180 and −162 bp from the transcription start site based on reporter gene expression. The underlined sequence (CAGA) is a consensus binding-site for Smad proteins. Luciferase activity was measured as described in Materials and Methods. (B) 3BRE is a luciferase fusion construct with a triple BRE repeat. The 3BRE construct (20 pg/embryo) was co-injected with *DN-BR* or *BMP-4* (each 0.5 ng/embryo) into 2-cell stage embryos. Animal caps were dissected at stage 8.5 and incubated until stage 11 in 0.5X MBS for measurement of luciferase activity as described in Materials and Methods. (C) The -180 WT and -180 MT constructs (20 pg/embryo) were injected with or without *BMP-4* or *Smad1* (each 0.5 ng/embryo) into 2-cell stage embryos. -180 MT indicates a construct with mutated BRE. Luciferase activity was measured as described in Materials and Methods. The sequences underlined indicate alterations in the original sequences. Experiments were repeated three times using independent sample sets. Data are shown as mean ± SD.

To identify and analyze putative BREs in the 5′-flanking region of *PV.1A*, we first quantitated the promoter activities of our serial deletion mutants in the presence of BMP-4, finding that ten deletion constructs retaining at least 180 bp of sequence 5′ of the major transcription initiation site responded to BMP-4 signaling, while the activity of the -162 construct was completely abolished (Fig. 3A).

Interestingly, CAGA sequences, which comprise a portion of the BRE, can be found within the region between 180 and 162 bp upstream of the *PV.1A* transcription start site. We therefore further analyzed this putative BRE by site-directed mutagenesis of our -180 construct, as well as through a synthetic threefold BRE tandem repeat construct (3BRE). The 3BRE construct responded positively to BMP-4 and negatively to DN-BR (Fig. 3B); when CAGA was mutated to CCAA in the core BRE region of the -180 construct, response to BMP-4 and Smad1 was abolished as well (Fig. 3C). Furthermore, in electrophoretic mobility shift assays (EMSAs) we found a direct interaction of the 3BRE construct with Smad1 and Smad4 (Fig. S2A). Interestingly, Smad3 also bound to the BRE (Fig. S2B), indicating that this response element alone is insufficient to precisely specify Smad binding, which instead appears to necessitate other *cis*-acting elements.

We examined promoter fragments of differing length in extracts of uninjected animal cap explants by competition assay, using competitor probes to determine whether endogenous proteins exhibit specific interactions with core Smad-binding sequences. The appearance of bands in all labeled probes was dramatically diminished by competition with unlabeled wild-type -180 probe, but not wild-type -160 or -180 probe harboring a mutation in the CAGA region (Fig. S2C). These data suggest that *PV.1A* is directly regulated by BMP-4 signaling through binding of Smad1 and Smad4 to the BRE within its promoter.

### Binding of Smad mutants to the 5′-flanking region of *PV.1A*

We examined the association of the BMP-4 signaling molecules Smad1 and Smad4 with the minimal *PV.1A* promoter (180 bp upstream of the transcriptional start site) in response in BMP-4 stimulation, using the wild-type (-180 WT) and mutant (-180 MT) constructs. Expression of wild-type Smad1, Smad4, and Smad1(4SA) significantly increased *PV.1A* promoter activity in the -180 WT construct. In addition, co-injection of *Smad1* and *Smad4* further increased promoter activity of the -180 WT construct compared with either mRNA alone (Fig. S3A). Dominant-negative *Smad1* (*Smad1*(3SA), with the three serine residues in its C-terminal SSVS mutated to alanines); *Smad1*(3.4SA), generated from *Smad1*(3SA) by replacing the linker region with that of *Smad1*(4SA); and mutant Smad2 isoforms did not alter transcriptional activation of this minimal promoter (Fig. S3A, B). Furthermore, expression of the -180 MT construct, in which the CAGA sequences in BRE were altered to CCAA, was unaffected by all Smads (Fig. S3A, B). These results demonstrate that the CAGA sequences of the BRE in the minimal *PV.1A* promoter are critical and essential for the response to BMP-4 and that the -180 region contains the specific response region for BMP signaling mediated through Smad effectors.

### Oaz cooperates with BMP-4 signaling in the 5′-flanking region of *PV.1A*

**Figure 4:**
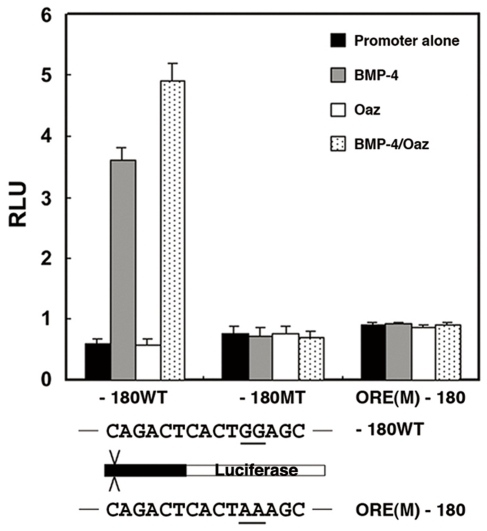
Oaz and BMP-4 cooperate to regulate *PV.1A* transcription. -180, -180 MT, and ORE(M)-180 constructs (20 pg/embryo) were injected with or without *BMP-4* (0.5 ng/embryo) and *Oaz* (0.1 ng/embryo) into the animal poles of 2-cell stage embryos. Animal caps were dissected from injected embryos at stage 8.5 and incubated until stage 11 in 0.5X MBS for measurement of luciferase activity as described in Materials and Methods. The sequences underlined depict alterations to the original sequences. Experiments were repeated three times using independent sample sets. Data are shown as mean ± SD.

A putative ORE lies in similar regions in the *PV.1A* and *Xvent-2* promoters (Fig. S1A). We therefore examined possible cooperativity of Oaz, a known transcriptional co-activator of *Xvent-2* family members [16], [22], with BMP-4. As expected, we observed additive reporter activity in animal cap explants on co-injecting *Oaz* and *Bmp-4* with the -180 promoter construct (Fig. 4). We then examined whether the putative ORE element could mediate this additive transcriptional induction. When the putative ORE sequence, TGGAGC, was mutated to TAAAGC in the -180 promoter construct, BMP-4 and Oaz cooperativity were completely abolished in luciferase reporter assays (Fig. 4). Notably, the response to BMP-4 and Oaz was also diminished when the BRE or ORE in the -180 promoter construct was mutated (Fig. 4). These data indicate that the BRE and ORE in the *PV.1A* promoter are both necessary for the appropriate transcriptional response to BMP-4 signaling.

### Xvent-2 enhances *PV.1A* promoter activity

**Figure 5:**
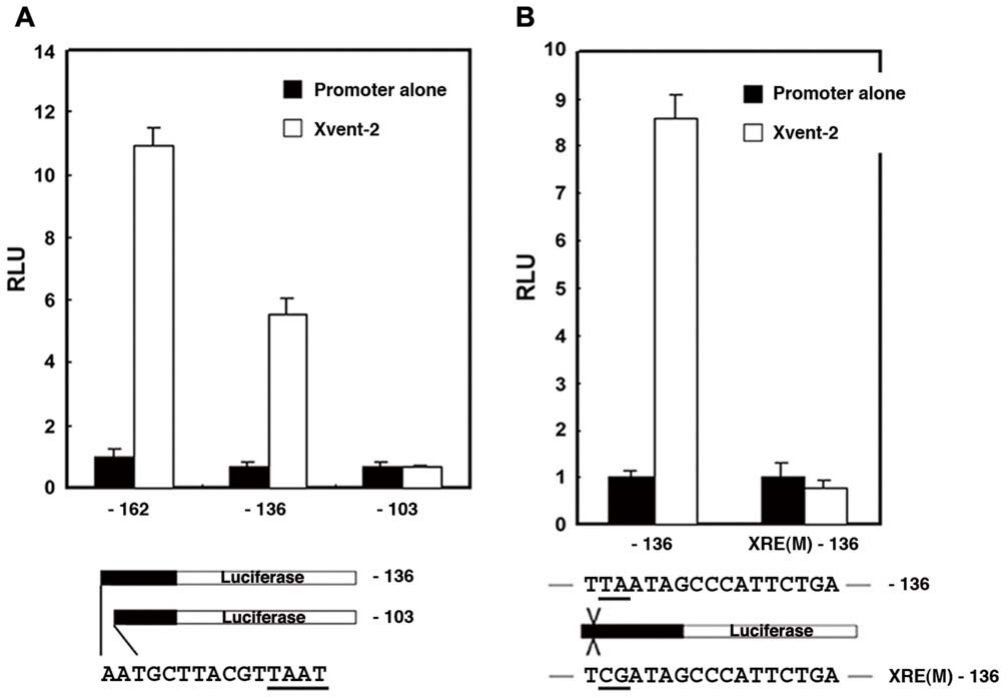
Positive regulation by Xvent-2 of the 5′-flanking region of *PV.1A*. (A) Serial-deletion mutant constructs (−162, −136, and −103; 20 pg/embryo) of the *PV.1A* promoter were co-injected with *Xvent-2* (0.5 ng/embryo) into the animal poles of 2-cell stage embryos. Animal caps were dissected from injected embryos at stage 8.5 and incubated until stage 11 in 0.5X MBS for measurement of luciferase activity (as described in Materials and Methods). A putative Xvent-2 binding site was detected between −136 and −103 bp from the major transcription initiation site. The underlined sequence, TAAT, is a consensus binding-site for homeobox proteins. (B) The −136 and XRE(M)-136 constructs were injected (20 pg/embryo) with or without *Xvent-2* (0.5 ng/embryo) at the animal poles of 2-cell stage embryos. Luciferase activity was measured as described in Materials and Methods. The sequences underlined indicate alterations to original sequences. Experiments were repeated three times using independent sample sets. Data are shown as mean ± SD.

*Xvent-1*, which, unlike *Xvent-2*, is not under direct control of BMP-4 signaling, can be transcriptionally up-regulated by Xvent-2 [21]. To identify the Xvent-2 response element (XRE) in the *PV.1A* promoter, we co-injected constructs containing serial deletion mutants of the *PV.1A* promoter driving reporter-gene expression into 2-cell stage embryos with *Xvent-2* mRNA. We found that the Xvent-2 response region in the *PV.1A* promoter differed from the BRE (Fig. 5A): Xvent-2 mediated transcriptional induction of the -136 construct was abrogated in the -103 construct. Notably, the region between -136 and -103 bp contained a TAAT homeobox-protein binding motif, and reporter activity of the -136 construct increased approximately 10-fold in the presence of Xvent-2 (Fig. 5A).

To determine whether this TAAT motif constituted the core XRE critical for Xvent-2 mediated *PV.1A* expression, we co-injected point mutant constructs [XRE(M) -136] into 2-cell stage embryos with *Xvent-2* mRNA. In the XRE(M) -136 construct-injected embryos, promoter activity was not induced by Xvent-2, while the WT -136 construct retained activity (Fig. 5B). These results indicate that the XRE lies between -136 and -103 bp of the *PV.1A* promoter and that the TAAT sequence is the core *cis*-element for Xvent-2 binding and transcriptional transctivation.

### Dorsal-specific transcription factors decrease *PV.1A* promoter activity

To identify negative *trans*-acting elements within the *PV.1A* promoter, mRNAs encoding the dorsal- and neural-specific transcription factors *Goosecoid* and *AP-1* were co-injected with the -2525 *PV.1A* promoter construct into 2-cell stage embryos. Both Goosecoid and AP-1 significantly down-regulated *PV.1A* promoter activity (Fig. S4). These results suggest that dorsal- and neural-specific transcription factors (particularly Goosecoid) are involved in repression of the *PV.1A* promoter in the dorsal mesoderm and neural ectoderm. In addition, the downregulation of *PV.1A* promoter activity by AP-1 implies the existence of uncharacterized cross-talk among several signaling pathways, including BMP-4, FGF, and activin.

## Discussion

Here, we examined the role of BMP-4 signaling on transcriptional regulation of *PV.1A*, a BMP-4 target gene encoding a transcription factor instrumental in the formation of ventral mesoderm during early *Xenopus* development. While BMP-4 was not previously thought to directly impact the expression of *Xvent-1* family members, we find that BMP-4 signaling plays a substantial role in modulation of *PV.1A* expression and that *Xvent-2*, in contrast, occupies a subsidiary role.

We first investigated whether *PV.1A* was under direct regulation by BMP-4. Our findings indicated *PV.1A* transcription in the presence of CHX and BMP-4, suggestive of a direct contribution of BMP-4 signaling to gene induction. Additionally, co-injection of *Xvent-2* into embryos with DN-BR mediated abrogation of BMP-4 signaling could not rescue reduced *PV.1A* expression, suggesting that Xvent-2 alone is insufficient for full *PV.1A* expression. Further, the decrease in *PV.1A* expression caused by DN Xvent-2 was mitigated by constitutively active Smad1, suggesting that basal *PV.1A* expression does not require Xvent-2, and *PV.1A* expression indeed appeared much more sensitive to BMP-4 signaling than to Xvent-2. In a previous study, we established slight induction of *Xvent-2* expression (∼2-fold) by BMP-4 signaling [16], in contrast to our current demonstration of BMP-4's dramatic effect on *PV.1A* expression (≥7-fold induction).

We reasoned that analysis of the *PV.1A* promoter would elucidate the mechanics of its combinatorial regulation. We isolated the *PV.1A* genomic locus, including the 5′-flanking region (−2525 bp), and generated serial promoter deletions to investigate their several contributions to gene induction. The region 180 bp upstream of the major transcriptional initiation site had full reporter activity in response to BMP-4 signals; and within this region, putative binding sites for Smad family members and Oaz were necessary and sufficient to mediate the effects of BMP-4 signaling. Additionally, we detected a cooperative interaction between these factors in promoting *PV.1A* transcription. Notably, the BRE in the *PV.1A* promoter was insufficient for Smad specificity, which was achieved only in the context of an ORE proximal to a BRE.

Using serial-deletion and point-mutation containing promoter fragments to drive reporter-gene expression, we determined that the *PV.1A* promoter contained a positive-regulatory *cis*-element and an XRE within 399 bp upstream of the major transcriptional initiation site: this latter element merely potentiated BMP-4 signaling for full transcriptional activation of *PV.1A*. We also investigated the negative regulation of the *PV.1A* promoter via co-injection of the dorsal-specific transcription factors AP-1 and Goosecoid, determining that each can repress *PV.1A* transcription. Our results suggest that Smads, Oaz, and Xvent-2 stimulate *PV.1A* expression and that the *PV.1A* promoter harbors the unique characteristic of possessing multiple *cis*-acting response elements for its regulation, including direct, indirect, co-activator, and negative response elements, to regulate the early embryonic development of *Xenopus laevis*.

A comparison of *PV.1A* 5′ sequence to promoters of other *Xvent* family members, including *Xbr-1a/Xvent-2* and *Xvent-2B*, found minor conservation of regulatory motifs, principally in the BRE and ORE, while *PV.1A* and *Xvent-1* share considerable sequence identity upstream of their transcriptional start sites (Fig. 2A). *Xvent-1*, however, is an indirect target of BMP-4 signaling [21]. Xvent-2 is not competent to rescue *PV.1A* expression in the absence of BMP-4 signal transduction; and the induction of each depends on BMP-4, suggesting that although the *PV.1A* and *Xvent-1B* promoters are nearly identical and even they belong to same family, their expression is differentially regulated. For instance, other regulatory elements, such as introns, may influence their transcription.

The zinc-finger protein Oaz is a DNA-binding co-factor that associates directly with the MH2 domain of Smad1 *in vitro* [22]. A complex of Smad1, Smad4, and Oaz binds to a BRE in the *Xvent-2* promoter, to enhance *Xvent-2* transcription upon BMP-4 stimulation of cultured cells [22]. We likewise find this requirement for Oaz in induction of the *PV.1A* promoter. Our data demonstrate a critical role for the BRE in the *PV.1A* BMP-4 response, as transcriptional activation is quelled by its mutation; the ORE imparts Smad specificity. 3BRE sequence bound Smad1, Smad4, and Smad3, thereby implying that the BRE is insufficient to confer Smad specificity, which might require other *cis*-acting elements. Although we did not examine their interaction, Oaz and Smads interact directly [22]. The ORE might mediate *PV.1A* transcription through interactions between Oaz, Smad1, and Smad4 at the BRE.

As shown in Fig. 1A, Xvent-2 failed to rescue the *DN-BR* induced reduction in *PV.1A* transcripts, demonstrating that *PV.1A* is a direct target of BMP-4 signaling and that its expression requires BMP-4 signaling. Xvent-2 has dual activities during early embryonic development. In the dorsal embryo, Xvent-2 is a repressor, but on the ventral side, an activator. We explored the function of Xvent-2 on in the ventral embryo and identified a putative XRE in the *PV.1A* promoter. Recently, the *Xvent-1B* promoter was shown to be activated by cooperation between Xvent-2 and GATA-2 [15], and several putative binding elements were identified; however, a specific *cis*-acting element for Xvent-2 was not identified in the *Xvent-1B* promoter. We identified a specific XRE between −136 and −103 bp in the *PV.1A* promoter, but this motif is not sufficient for complete transcriptional activation of *PV.1A* and may function primarily as an enhancer of BMP-4 signaling.

In the developing *Xenopus* embryo, ventral-specific genes are repressed within dorsal or organizer tissues. This repression may be mediated though several mechanisms. First, activin-like signaling on the dorsal side induces the expression of BMP-4 antagonists such as Chordin, Noggin, and Follistatin. These antagonists are secreted extracellularly and bind to BMP-4, preventing it from accessing its receptor. In the absence of BMP-4 signaling, its downstream target genes cannot be transcribed.

Alternatively, ventral genes are repressed dorsally by dorsal-specific proteins such as Goosecoid and AP-1 through binding of dorsal-specific repressors to consensus sites in promoters of ventral-specific genes. *PV.1A*, but not *Xvent-2*, is repressed by Goosecoid (data not shown). We are currently biochemically investigating the mechanisms by which *PV.1A* is repressed within dorsal and organizer tissues using the *PV.1A* promoter to identify negative *cis*-acting elements that are bound and regulated by dorsal-specific proteins.

In this study, we investigated the transcriptional regulation of *PV.1A* and identified *cis*-acting elements, including BRE, ORE, and XRE, in its promoter sequences. Additionally, we found that the dorsal-specific transcription factors Goosecoid and AP-1 exert negative effects on *PV.1A* reporter activity through BMP-4 signaling. Our results indicate that the complex transcriptional control exerted over downstream effectors of BMP-4 occurs through interplay between positive and negative regulators of transcription.

## Supporting Information

Figure S1 Bioinformatics analysis of the 5′-flanking region of *PV.1A*. (A) Comparison among the 5′-flanking sequences of *PV.1A*, *Xbr-1a/Xvent-2*, and *Xvent-2B* indicates a high level of identity within BMP-4 response elements. BRE, ORE, and XRE indicate BMP-4, Oaz-, and Xvent-2 response elements, respectively. (B) The schematic diagram illustrates serial-deletion promoter constructs. Serially deleted and site-directed mutant constructs were subcloned into the pGL2-basic plasmid. Arrowheads indicate positions of site-directed mutagenesis.
(JPG)

Figure S2 BRE binding assays. The BMP-4 response element was confirmed by EMSA using probes against triple-repeat BRE (A, B) or the TATA-box region of the promoter (C, D). The protein extracts indicated were obtained from *in vitro* translated proteins (A, B) or uninjected animal caps (C, D). Unlabeled competitor probe was added at 100-fold excess (C) or the concentration indicated (D). Asterisks indicate specific bands.
(JPG)

Figure S3 Smad binding within the 5′-flanking region of *PV.1A*. (A, B) The -180 WT construct (20 pg/embryo) were co-injected with the RNAs indicated (0.5 ng/embryo) into 2-cell stage embryos. Animal caps were dissected from injected embryos at stage 8.5 and incubated until stage 11 in 0.5X MBS for measurement of luciferase activity as described in Materials and Methods. Experiments were repeated three times using independent sample sets. Data are shown as mean ± SD.
(JPG)

Figure S4 Repressive effects of dorsal-specific protein binding to the 5′-flanking region of *PV.1A*. The -2525 *PV.1A* promoter construct (20 pg/embryo) was co-injected with *BMP-4* (0.5 ng/embryo) and *Goosecoid* (0.1 ng/embryo) or *AP-1* (0.5 ng/embryo) into 2-cell stage embryos. Animal caps were dissected from injected embryos at stage 8.5 and incubated until stage 11 in 0.5X MBS for measurement of luciferase activity as described in Materials and Methods. Experiments were repeated three times using independent sample sets. Data are shown as mean ± SD.
(JPG)
