# Supplementary figures and images for: Direct Response Elements of BMP within the PV.1A Promoter Are Essential for Its Transcriptional Regulation during Early Xenopus Development
Source: PLoS One. 2011 Aug 3;6(8):e22621. doi: 10.1371/journal.pone.0022621 (PMC3153937; doi:10.1371/journal.pone.0022621)

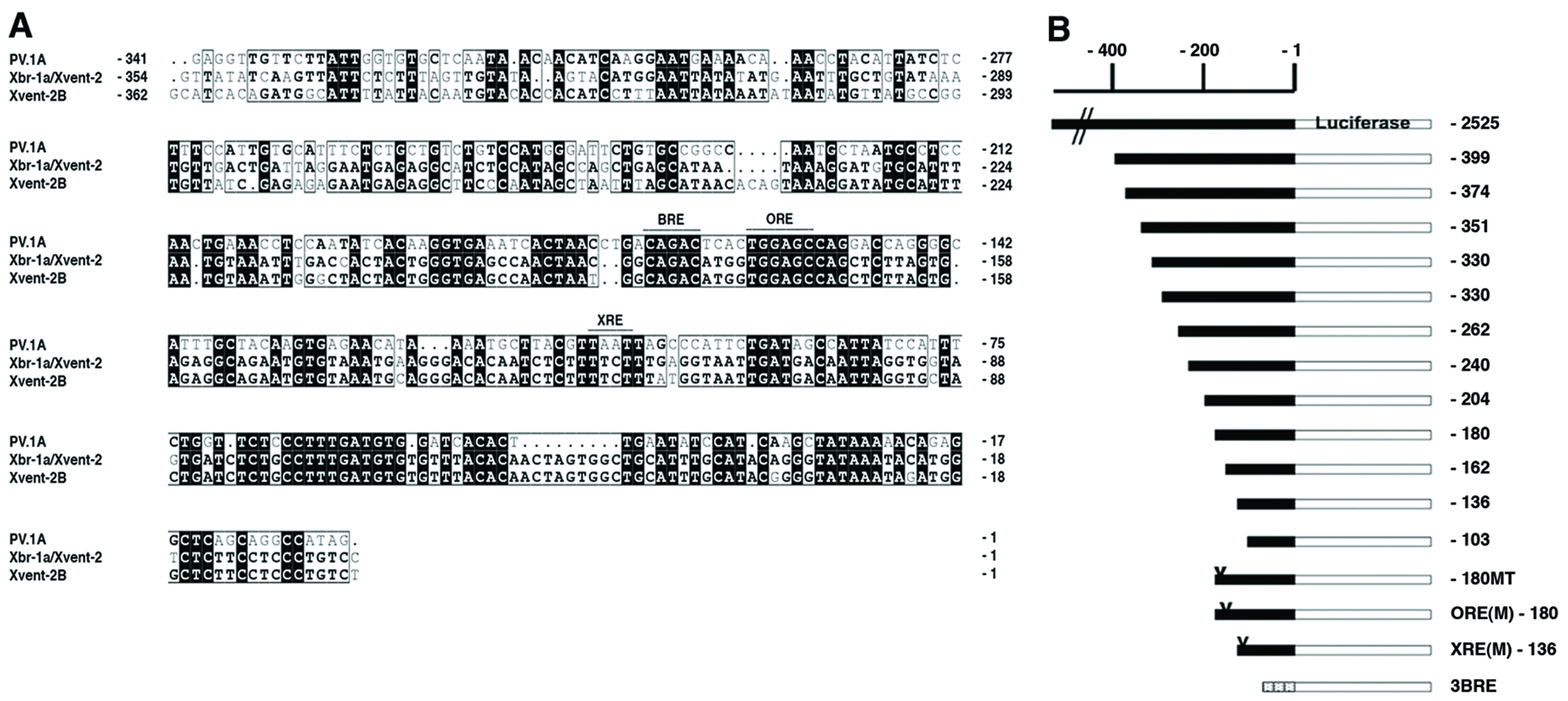

Supplement: Figure S1 — Bioinformatics analysis of the 5′-flanking region of PV.1A. (A) Comparison among the 5′-flanking sequences of PV.1A, Xbr-1a/Xvent-2, and Xvent-2B indicates a high level of identity within BMP-4 response elements. BRE, ORE, and XRE indicate BMP-4, Oaz-, and Xvent-2 response elements, respectively. (B) The schematic diagram illustrates serial-deletion promoter constructs. Serially deleted and site-directed mutant constructs were subcloned into the pGL2-basic plasmid. Arrowheads indicate positions of site-directed mutagenesis. (JPG) [file pone.0022621.s001.jpg]

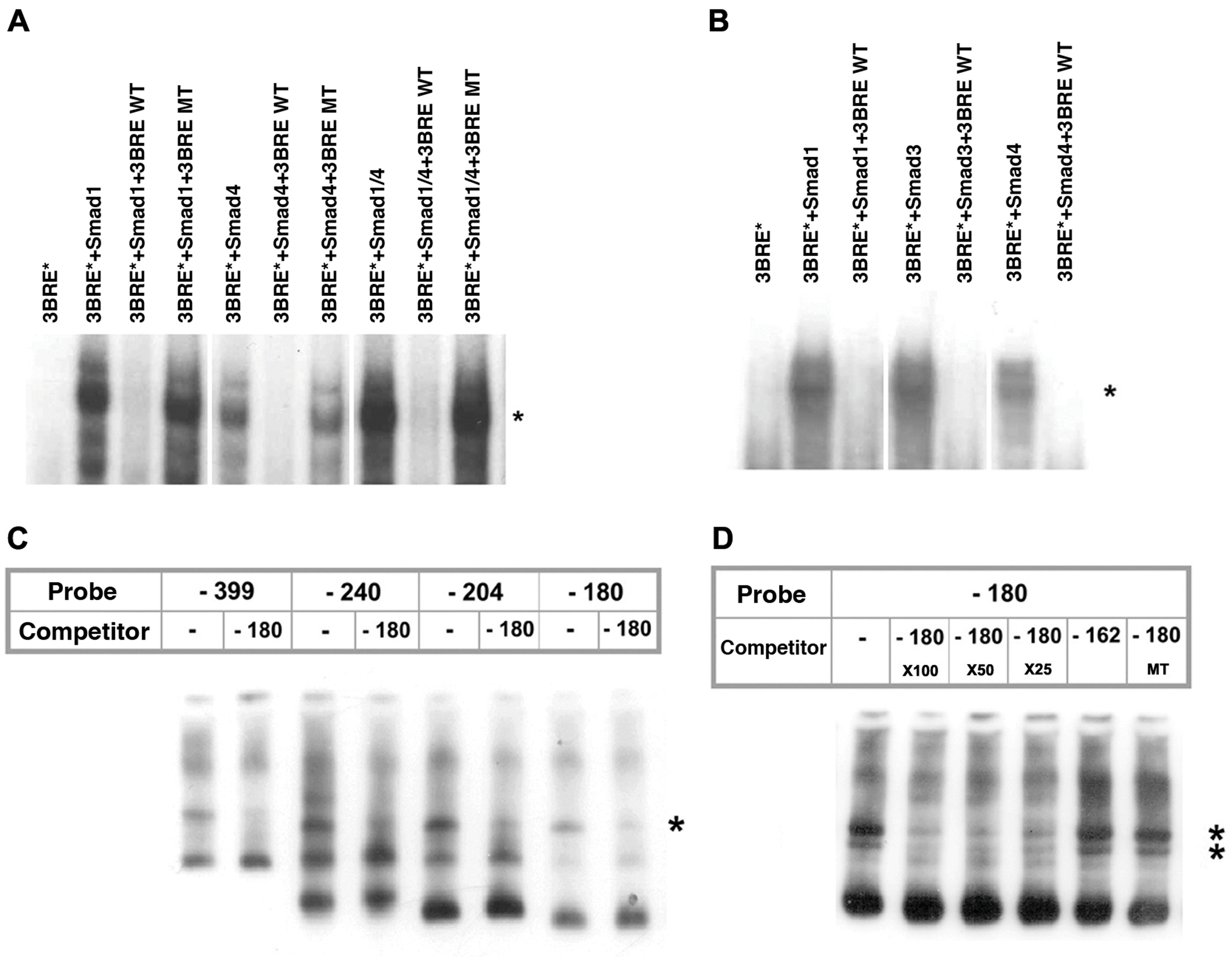

Supplement: Figure S2 — BRE binding assays. The BMP-4 response element was confirmed by EMSA using probes against triple-repeat BRE (A, B) or the TATA-box region of the promoter (C, D). The protein extracts indicated were obtained from in vitro translated proteins (A, B) or uninjected animal caps (C, D). Unlabeled competitor probe was added at 100-fold excess (C) or the concentration indicated (D). Asterisks indicate specific bands. (JPG) [file pone.0022621.s002.jpg]

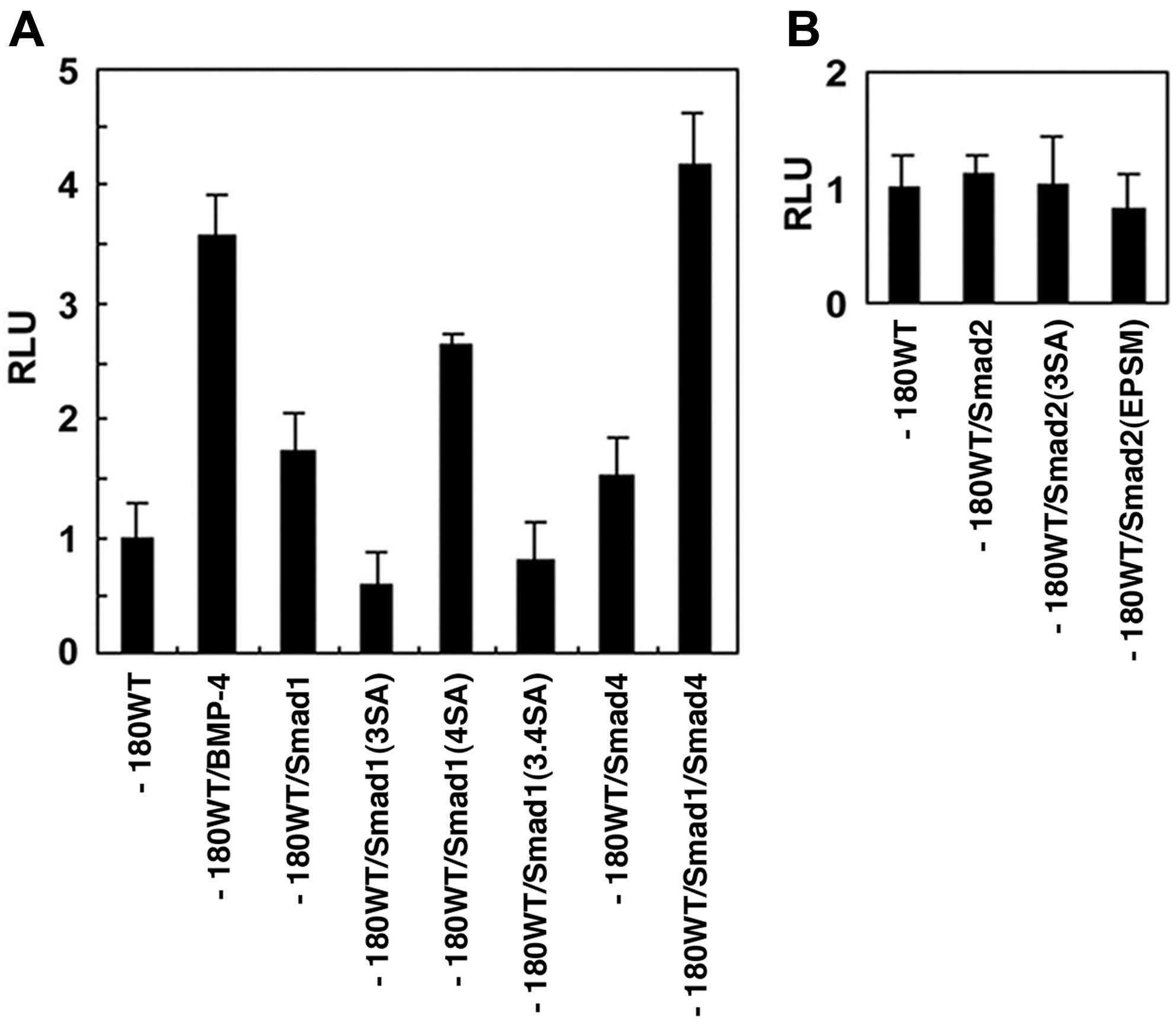

Supplement: Figure S3 — Smad binding within the 5′-flanking region of PV.1A. (A, B) The -180 WT construct (20 pg/embryo) were co-injected with the RNAs indicated (0.5 ng/embryo) into 2-cell stage embryos. Animal caps were dissected from injected embryos at stage 8.5 and incubated until stage 11 in 0.5X MBS for measurement of luciferase activity as described in Materials and Methods. Experiments were repeated three times using independent sample sets. Data are shown as mean ± SD. (JPG) [file pone.0022621.s003.jpg]

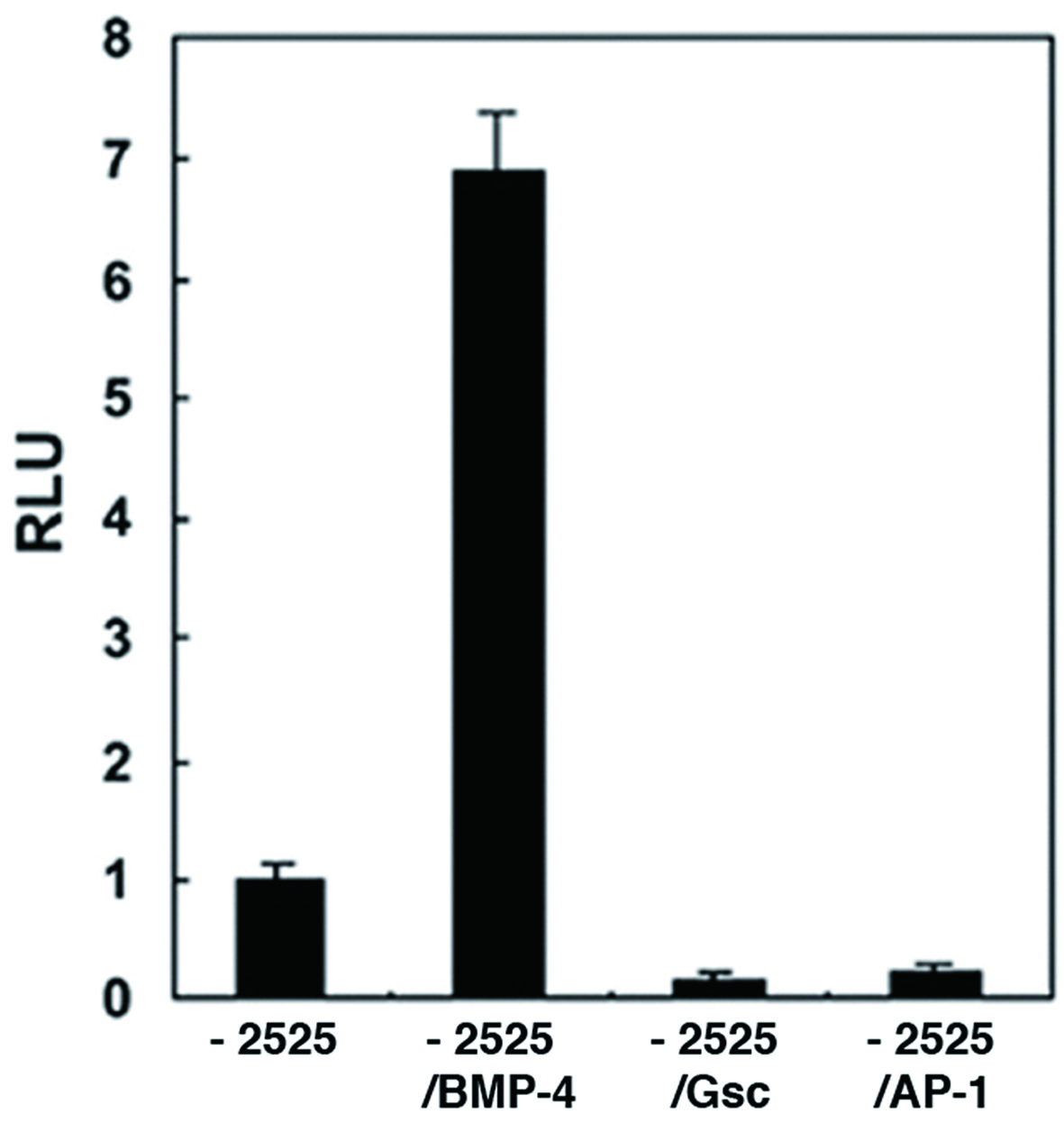

Supplement: Figure S4 — Repressive effects of dorsal-specific protein binding to the 5′-flanking region of PV.1A. The -2525 PV.1A promoter construct (20 pg/embryo) was co-injected with BMP-4 (0.5 ng/embryo) and Goosecoid (0.1 ng/embryo) or AP-1 (0.5 ng/embryo) into 2-cell stage embryos. Animal caps were dissected from injected embryos at stage 8.5 and incubated until stage 11 in 0.5X MBS for measurement of luciferase activity as described in Materials and Methods. Experiments were repeated three times using independent sample sets. Data are shown as mean ± SD. (JPG) [file pone.0022621.s004.jpg]
